# Pyrolytic Modification of Heavy Coal Tar by Multi-Polymer Blending: Preparation of Ordered Carbonaceous Mesophase

**DOI:** 10.3390/polym16010161

**Published:** 2024-01-04

**Authors:** Lei Zhang, Chunjiang Liu, Yang Jia, Yidan Mu, Yao Yan, Pengcheng Huang

**Affiliations:** 1 College of Geology and Environment, Xi’an University of Science and Technology, Xi’an 710054, China; 22209226097@stu.xust.edu.cn (C.L.); 23209226116@stu.xust.edu.cn (Y.M.); efqg011991@outlook.com (Y.Y.); 2 Key Laboratory of Coal Resources Exploration and Comprehensive Utilization, Ministry of Natural Resources, Xi’an 710021, China; 3 State Key Laboratory of Eco-Hydraulics in Northwest Arid Region, Institute of Water Resources and Hydro-Electric Engineering, Xi’an University of Technology, Xi’an 710048, China; 1200413058@stu.xaut.edu.cn; 4 Coal Geology Bureau of Ningxia Hui Autonomous Region, Yinchuan 750002, China; 13299505297@sohu.com

**Keywords:** copolymer, heavy coal tar, carbonaceous mesophase, ordered modification, graphitization degree

## Abstract

In order to achieve the high-value utilization of heavy tar for the production of enhanced-performance graphite foam carbon, the carbon mesophase was ready from the heavy component of low-temperature coal tar, and the coal tar was modified by styrene-butadiene-styrene (SBS), polyethylene (PE) and ethylene-vinyl-acetate (EVA) copolymers. The order degree of the carbonite mesophase was analyzed using a polarizing microscope test, Fourier transform infrared spectroscopy and X-ray diffraction to screen out the most suitable copolymer type and addition amount. Furthermore, the mechanism of modification by this copolymer was analyzed. The results showed that adding SBS, PE and EVA to coal tar would affect the order of carbonaceous mesophase; however, at an addition rate of 10.0 wt.%, the linear-structure SBS copolymer with a styrene/butadiene ratio (S/B) of 30/70 exhibited the optimal degree of ordering in the carbonaceous mesophase. Its foam carbon prepared by polymer modification is the only one that forms a graphitized structure, with d_002_ of 0.3430 nm, and the maximum values of Lc and La are 3.54 nm and 2.22 nm, respectively. This is because, under elevated pressure and high-temperature conditions, SBS underwent chain scission, releasing a more significant number of methyl and other free radicals that interacted with the coal tar constituents. As a result, it reduced the affinity density of heavy coal tar molecules, enhanced fluidity, promoted the stacking of condensed aromatic hydrocarbons and increased the content of soluble carbonaceous mesophase, ultimately leading to a more favorable alignment of the carbonaceous mesophase.

## 1. Introduction

Heavy coal tar is a byproduct obtained through the distillation and steam cracking of coal tar, with its asphalt content (boiling point above 360 °C) exceeding 50% [1]. It inherently contains a rich mixture of polycyclic aromatic hydrocarbons (PAHs), coke and volatiles, endowing it with significant value for high-value utilization [2]. The preparation of graphitized foam carbon represents a sustainable avenue for its utilization. Such carbon materials find widespread applications in metallurgy, construction, textiles, papermaking and transportation, exhibiting promising prospects [2,3,4]. The ordered nature of the carbonaceous intermediate phase formed during the pyrolysis of heavy tar is a crucial factor influencing the performance of graphitized foam carbon [5].

Brooks and Taylor discovered mesophase in the 1960s [6]. Afterward, it became a significant intermediate for the preparation of carbon fibers [7,8,9], carbon-based mesophase foam carbon [10,11,12], ultra-high specific surface area activated carbon [13,14], carbonaceous electrodes [15,16] and other composite materials, which are widely used in aerospace [17,18], energy fuel [19,20], medical [21] and other fields. The carbonaceous mesophase, or mesophase carbon microspheres, is a material form between liquid-phase and solid-phase crystals [6]. Previous studies have shown that the carbonaceous mesophase is an essential precursor for the formation of graphitized structures. In addition, its design and molecular arrangement affect the system of graphitized carbon-based materials [22,23]. Therefore, the ordered state of the mesophase is a vital prerequisite for preparing graphitized foam carbon.

Graphitization represents the transition from amorphous to crystalline state, signifying a transformation at the microscopic level where the carbon atom arrangement evolves from disorderly to orderly [24,25]. The carbonaceous mesophase contains various aromatic formats with many sp^2^-hybridized carbon atoms, and the aromatic molecules are highly analogous to the ortho-hexagonal arrangement of carbon atoms in the graphite lattice. Therefore, the mesophase has the potential to be transformed into graphitic carbon materials [26,27,28]. In recent years, there has been research on adjusting mesophase to enhance material properties. For instance, treating stable fibers with a boric acid solution enables the preparation of mesophase asphalt-based carbon fibers with varying boron concentrations [29]. Additionally, laser-induced graphitization of mesophase asphalt-based carbon fibers can enhance their electrical conductivity [30]. Consequently, the ordered preparation of mesophase presents a challenging yet valuable area of investigation.

Numerous studies have demonstrated that the introduction of certain copolymers into the reaction system allows for intervention in the formation of mesophase nuclei, their growth or enlargement and the fusion process. Consequently, this can alter the morphology and microstructure of the original mesophase [31,32,33,34]. Among them, styrene-butadiene-styrene (SBS), polyethylene (PE) and ethylene-vinyl-acetate (EVA) were the more frequent copolymers. Cheng et al. [35] used waste SBS to modify asphalt and found that the addition of waste SBS significantly increased the methylene content in cokes. The research improved the thermal stability of the co-carbonated product. Cheng et al. [36] prepared asphalt modified with waste polyethylene (WPE)/styrene-butadiene-styrene block copolymer (SBS) and nano-CaCO_3_ with different ratios using the melt blending method and found that the needle penetration of the modified asphalt increased and the ductility decreased with the increase in the WPE admixture. In addition, the softening point of the blends increased with the increase in WPE/SBS content. Li et al. [37] studied carbon fiber precursors based on PE-g-PAH graft copolymers. This approach reduced production costs and improved the compatibility between copolymers and free asphalt molecules in the precursor. This compatibility enhancement facilitated a high carbon yield during the thermal conversion process, ultimately resulting in the production of high-strength carbon fibers. Dou et al. [38] incorporated varying amounts of ethylene-vinyl acetate copolymer (EVA) into a thermoplastic polyurethane (TPU) matrix and observed that EVA modified the phase morphology and crystalline structure of the blend system, increasing the melt viscosity and crystallinity of the blend system.

The extensive structure and diverse chemical elements ensure the plasticity of heavy coal tar, rendering it attractive for polymer modification. On the other hand, the complexity of the layered orientation and texture in the carbonaceous intermediate phase poses a challenge to the controlled preparation of this intermediate phase. However, there is currently limited research that systematically compares the ordered structures of SBS-, PE- and EVA-modified carbonaceous intermediate phases based on graphitization degree. Such a comparison is crucial for the improved preparation of graphitized foam carbon.

In this study, carbonaceous mesophase was prepared from low-temperature heavy coal tar as the raw material, and coal tar was modified with SBS, PE and EVA to enhance the degree of orderliness of the carbonaceous mesophase. At the same time, the study elucidated the influence of SBS structure, blending ratio and content; PE density and content; and EVA with varying vinyl acetate (VA) content on the changes in carbonaceous mesophase content and structure. Lastly, a comprehensive analysis was conducted from various perspectives, including microstructure, crystal peak pattern and functional groups, to elucidate the impact of copolymers on the structural order of the carbonaceous mesophase. The results of this experiment contribute to the high-value utilization of heavy coal tar, promoting sustainable energy production and providing guidance for the subsequent production of high-performance foam carbon materials.

## 2. Materials and Methods

### 2.1. Materials

The raw material selected for this experiment was heavy coal tar derived from a low-temperature coal tar production facility in Yulin City, Shaanxi Province, China. The composition of the raw coal tar is shown in Table 1. It should be noted that the QI value is relatively small and can be disregarded.

The distribution of heavy tar fractions is presented in Table 2.

### 2.2. Experimental Reagent

The selected modifiers, SBS-1301 and SBS-4303, were sourced from Yanshan Petrochemical, Beijing, China. At the same time, SBS-YH-801, SBS-791-H and SBS-792 were obtained from Yueyang Baling Petrochemical, Yueyang, Hunan Province, China, as detailed in Table 3 regarding their properties.

The modifier PE selected in the experiments was from Sinopec, Beijing, China. Its properties are shown in Table 4.

The modifiers EVA selected in the experiments were all from Sinopec, Beijing, China. Their properties are shown in Table 5.

GAOPIN-G00548 (Kunshan Gaopin Precision Instrument Co., Ltd., Suzhou, Jiangsu Province, China) was chosen as the epoxy resin crystal glue for the cold mounting of metallographic specimens required in the experiment.

### 2.3. Sample Characterization

#### 2.3.1. Optical Structure Analysis

The mesophase asphalt produced by the heat transformation reaction was placed in a mold, injected with a cold-embedding agent, demolded after curing and subsequently subjected to rough grinding and polishing. Finally, it was observed under a Leica DM750P optical microscope (Leica, Germany) to examine the anisotropic mesophase morphology.

#### 2.3.2. FT-IR Analysis

The VERTEX 70 Fourier-transform infrared (FT−IR) spectrometer (Bruker, Germany) was used. A suitable amount of the sample was prepared for infrared testing analysis within the spectral range of 4000 cm^−1^ to 400 cm^−1^. The spectra were collected with 28 scans and a spectral resolution of 0.4 cm^−1^.

#### 2.3.3. XRD Analysis

The analysis involved the use of the XD-3 X-ray diffractometer (Beijing Puxi General Instruments Co., Ltd., Beijing, China) for polycrystalline X-ray diffraction, solid sample phase analysis, trace phase analysis and low-angle diffraction analysis of the samples. Operational conditions included an initial angle of 5°, a final angle of 80°, a step width of 0.02, a wavelength of 1.54056 (using a Co Kα radiation source) and an operational voltage of 36 kV with a current of 20 mA.

Utilizing MDI Jade 6 software for sample XRD pattern analysis, crystallographic data were calculated using the following relevant formulas:

(1) $$\begin{array}{c} Lc=\frac{0.89\lambda }{\beta \cos\theta } \end{array}$$

(2) $$\begin{array}{c} La=\frac{1.84\lambda }{\beta \cos\theta } \end{array}$$

(3) $$\begin{array}{c} d_{002}=\frac{\lambda }{2\sin\theta } \end{array}$$

(4) $$\begin{array}{c} g=\frac{0.3440\;-d_{002}}{0.3440\;-0.3354} \end{array}$$

where Lc was microcrystal stacking thickness (nm), λ was incident wavelength (0.15406 nm), β was half-peak width, La was microcrystal size (nm), θ was (002) crystallographic diffraction angle, 0.3440 nm was entirely non-graphitized carbon material (002) crystalline surface layer spacing, and 0.3354 nm was ideal single-crystal graphite layer spacing. d_002_ was calculated by XRD mapping (002) graphite characteristic diffraction peaks combined with the aid of MDI Jade 6 software.

### 2.4. Experimental Protocol

(1) Preparation of Carbonaceous Mesophase through Copolymer-Modified Heavy Coal Tar

We placed 50 g of heavy coal tar in a beaker and heated it in a water bath to 70 °C. Then, 30 g of the heat-treated heavy coal tar was weighed and heated to 170 °C with a heated magnetic stirrer at 150 rpm. Different SBS (structure, block ratio, addition amount), PE (density, addition amount) and EVA (addition amount, VA content) were set to be added into it. The rotation speed was then adjusted to 300 rpm, and the modification process was conducted at a constant temperature of 170 °C for 1 h. After sealing the modified coal tar mixture in a high-temperature, high-pressure tubular furnace, it was continuously purged with N_2_ for 2–3 min at a pressure of 1.0 MPa and left to stand for 15 min in the constant-temperature zone. Suppose there is no significant change in the pressure reading. In that case, it indicates that the high-temperature, high-pressure tubular furnace has good gas tightness. Subsequently, the temperature ramping program can be initiated. The temperature was raised to the final temperature of 400 °C over a period of 130 min, followed by isothermal heating for 12 h. After completing the heating process, the sample was removed and stored for characterization analysis once the high-temperature, high-pressure tubular furnace naturally cooled to room temperature.

(2) Evaluation system of carbonaceous mesophase ordination

In the study of carbon mesophase, polarizing microscopy was most often used to observe carbon mesophase. In the polarized optical micrographs, the brightest areas corresponded to the anisotropic regions, and the content of the mesophase was determined by the proportion of the anisotropic surface area [39]. While the polarizing microscope enables the observation of the microstructure of the mesophase and the assessment of its optical anisotropy and formation, it does not provide a direct means to intuitively determine the degree of orderliness [40,41,42]. In the field of graphite materials, the degree of graphitization is typically used to assess the level of carbon arrangement orderliness in the material [43,44]. Lc, La and d_002_, three structural parameters, were commonly utilized as crucial indicators for the structure of carbonaceous mesophases [45,46]. The calculation of the graphitization degree was based on d_002_, which was the carbon (002) crystalline spacing in the XRD test pattern of carbon material, and the (002) crystalline spacing of perfect graphite was 0.3354 nm. Through the graphitization degree evaluation criteria, the d_002_ value was proposed to be used as the evaluation criterion for the degree of carbon mesophase orderliness. The discount nearer to the d_002_ value of 0.3448 nm for carbon materials with graphitized structure indicated a better degree of the orderliness of the mesophase of the carbonaceous material.

## 3. Results and Discussion

### 3.1. Analysis of Coal Tar Components

**Figure 1:**
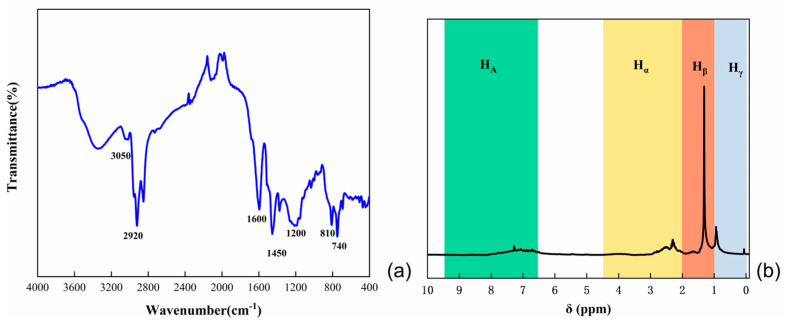
(**a**) Infrared spectra of coal tar; (**b**) ^1^H−NMR spectra of coal tar.

The analysis of natural coal tar by infrared chromatography is shown in Figure 1a. In the vicinity of 3050 cm^−1^, vibration absorption peaks corresponding to C–H bonds on benzene rings were observed. The peak at 1600 cm^−1^ indicated the vibration absorption of C=C bonds in aromatic rings, while peaks near 670 cm^−1^ were attributed to the vibration absorption of carbon-hydrogen substituent structures on benzene rings. The presence of saturated methylene C–H vibration absorption peaks was observed at 2920 cm^−1^, and C–H vibration absorption peaks of methyl groups were evident in the vicinity of 1450 cm^−1^. The stretching vibration peak of –OH groups was observed around 3430 cm^−1^, and absorption peaks at 740 cm^−1^ and 810 cm^−1^ were associated with out-of-plane bending vibrations of aromatic molecules. Consequently, the coal tar feedstock predominantly consisted of alkyl side chains, aliphatic hydrocarbons, PAHs and benzene ring structures.

^1^H-NMR analysis of coal tar raw materials is shown in Figure 1b. Due to the chemical shift of aromatic hydrogen (HA) in the range of 6.5 to 9.5, the chemical shift of aromatic side chain α hydrogen (H_α_) falls within the range of 2.0 to 4.5, the chemical shift of aromatic side chain β hydrogen (H_β_) is in the range of 1.0 to 2.0, and the chemical shift of aromatic side chain γ hydrogen (H_γ_) is in the range of 0 to 1.0. Combining the hydrogen distribution, it can be inferred that coal tar has a lower content of polycyclic aromatic hydrocarbons and a higher proportion of side chains and predominantly consists of α-methyl and α-methylene fatty side chains.

### 3.2. SBS-Modified Carbonaceous Mesophase

#### 3.2.1. Different SBS Structure Modified Carbonaceous Mesophase

**Figure 2:**
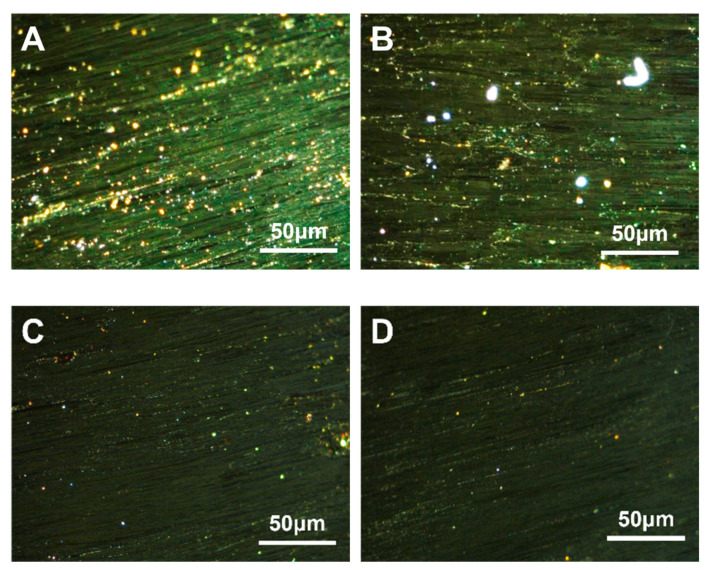
Polarization diagrams of carbonaceous mesophase prepared with SBS with different structures ((**A**)—MPS_1301_, (**B**)—MPS_791_, (**C**)—MPS_801_, (**D**)—MPS_4303_).

SBS is a triblock copolymer composed of styrene and butadiene. Depending on their arrangement, these two compounds can be categorized into linear and radial structures [47]. The structural formulae are {[CH_2_−CH(C_6_H_5_)]_n_− [CH_2_–CH=CH–CH_2_]_m_}_4_ and [CH_2_–CH(C_6_H_5_)]_n_– [CH_2_–CH=CH–CH_2_]_m_– [CH(C_6_H_5_)–CH_2_]_n_, respectively. The carbonaceous mesophase made from heavy coal tar with 4.0 wt.% of SBS-1301, SBS-791-H, SBS-YH-801 and SBS-4303 was noted as MPS_1301_, MPS_791_, MPS_801_ and MPS_4303_, respectively. The polarized images, magnified 500× *g*, are shown in Figure 2.

It could be found that the carbonaceous mesophase prepared by the modification of linear SBS was larger and generated more, mainly in the mosaic type. The molecular weight of radial SBS was greater than that of linear SBS; linear SBS could break more methyl radicals in the heating process to promote the thermal condensation reaction of the mesophase formation. Similarly, linear SBS with a more straightforward molecular structure was easier to fuse with coal tar molecules, which was conducive to promoting mesophase formation [48,49].

#### 3.2.2. Modified Carbon Mesophase with Different SBS Block Ratios

**Figure 3:**
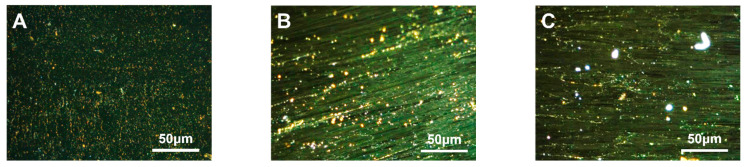
Polarization diagrams of carbonaceous mesophase prepared with SBS with different block ratios ((**A**)—MPS_792_, (**B**)—MPS_1301_, (**C**)—MPS_791_).

The carbonaceous mesophase made from heavy coal tar with 4.0 wt.% SBS-792, SBS-1301 and SBS-791-H was noted as MPS_792_, MPS_1301_ and MPS_791_. The polarization image after 500× *g* magnification is shown in Figure 3.

Upon observing Figure 3, it was found that the density and morphology of the carbonaceous mesophase prepared through SBS modification with a segment ratio of 30/70 (S/B) were superior. In Figure 3B, it was shown that an embedded-type structure appeared in the mesophase, indicating that the size of the mesophase was larger. Simultaneously, it was observed that the carbonaceous mesophase prepared from sample MPS_1031_ exhibited a distinct streamlined mesophase. Moreover, the streamlined texture was beneficial for spinning the mesophase into carbon fibers [50]. During the initial heating process of the pyrolysis–polymerization reaction, SBS with a segment ratio of 30/70 decomposed into more benzene-ring-type radicals. These radicals underwent specific cross-linking reactions with the benzene-ring-type radicals generated from coal tar pyrolysis, thereby facilitating the formation of the mesophase.

#### 3.2.3. Modified Carbonaceous Mesophase with Different SBS-1301 Additions

**Figure 4:**
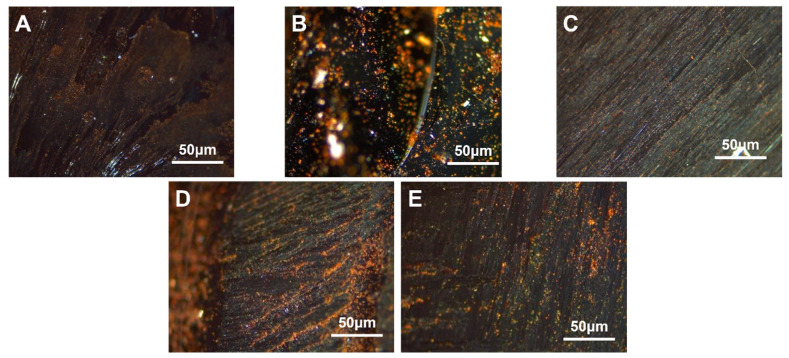
Polarization diagrams of carbonaceous mesophase prepared with different SBS-1301 additions (**A**)—MPS_1301-2_, (**B**)—MPS_1301-4_, (**C**)—MPS_1301-6_, (**D**)—MPS_1301-8_, (**E**)—MPS_1301-10_).

The carbonaceous mesophase made from coal tar with 2.0 wt.%, 4.0 wt.%, 6.0 wt.%, 8.0 wt.% and 10.0 wt.% of SBS-1301 was noted as MPS_1301-2_, MPS_1301-4_, MPS_1301-6_, MPS_1301-8_ and MPS_1301-10_. The polarized images of the carbonaceous mesophase magnified at a factor of 500× *g* are illustrated in Figure 4.

From Figure 4, it was observed that as the dosage of SBS-1301 increased gradually, the content of the carbonaceous mesophase exhibited a trend of initially increasing and then decreasing. Among them, the SBS-1301 modification with a 4.0 wt.% addition yielded a larger size, higher content and better orderliness of the carbonaceous mesophase. When the SBS addition was too low, the degree of modification was minimal, resulting in a relatively lower content of the mesophase. Conversely, when the addition exceeded 4.0 wt.%, the incomplete reaction of the SBS-derived radicals led to a decrease in orderliness and content in the preparation of the carbonaceous mesophase.

### 3.3. PE-Modified Carbon Mesophase

#### 3.3.1. PE-Modified Carbon Mesophase with Different Density

**Figure 5:**
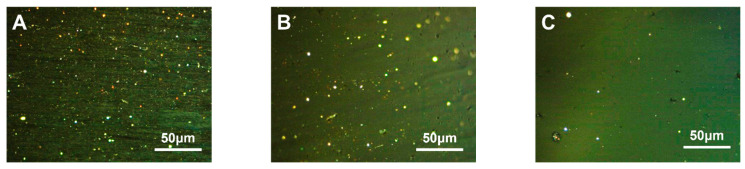
Polarization diagram of carbonaceous mesophase prepared by three kinds of PE density modification ((**A**)—MPP_1_, (**B**)—MPP_2_, (**C**)—MPP_3_).

PE granules with three different densities were used to prepare carbonaceous mesophase by mixing them with 4.0 wt.% of LDPE, LLDPE and HDPE. These resulting phases were denoted as MPP_1_, MPP_2_ and MPP_3_, respectively. The polarized images, magnified at a factor of 500× *g*, are depicted in Figure 5.

As shown in Figure 5A, the asphalt prepared as the mesophase with high-density polyethylene as a copolymer exhibited larger mesophase size and greater orderliness. It was likely due to the higher crystallinity of HDPE, ranging from 85% to 97%, compared to LDPE and LLDPE, which led to a more ordered molecular arrangement. Although its melting point at 130 °C was higher compared to the other two PE materials, its high crystallinity meant that, once the temperature exceeded the melting point, its molecular mobility increased significantly. This resulted in a sharp decrease in viscosity and improved flexibility, facilitating a more ordered flow in the mixed system and promoting the orderly growth of the carbonaceous mesophase [51,52]. Furthermore, the methyl and other side chains attached to the PE molecules participated in the polymerization reaction, promoting the formation of the mesophase and enhancing its structure and properties [39].

#### 3.3.2. Modified Carbonaceous Mesophase with Different HDPE Additions

**Figure 6:**
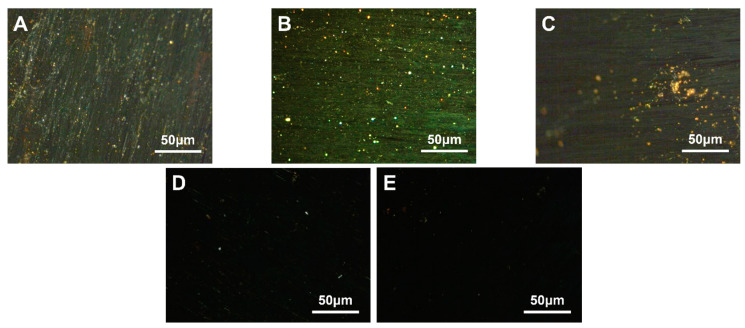
Polarization diagram of carbonaceous mesophase prepared with different addition amounts of HDPE ((**A**)—MPP_1-2_, (**B**)—MPP_1-4_, (**C**)—MPP_1-6_, (**D**)—MPP_1-8_, (**E**)—MPP_1-10_).

The carbonaceous mesophase made from heavy coal tar with 2.0 wt.%, 4.0 wt.%, 6.0 wt.%, 8.0 wt.% and 10.0 wt.% HDPE was noted as MPP_1-2_, MPP_1-4_, MPP_1-6_, MPP_1-8_ and MPP_1-10_, respectively. The polarized images, magnified 500× *g*, are shown in Figure 6.

The figure revealed that when the PE addition ranged from 2.0 wt.% to 4.0 wt.%, the content of the mesophase was relatively higher. However, at a 2.0 wt.% addition level, a more pronounced streamline texture was observed, indicating that PE at this additive level was more conducive to the growth of the mesophase. With an increase in the addition amount, the modified coal tar exhibited a decrease in ductility and an increase in softening temperature. When the addition amount exceeded 4.0 wt.%, a phase transition in the dispersed system of PE-modified coal tar might have occurred. The copolymer PE particles dispersed within the coal tar phase at lower addition amounts. As the addition amount increased to a certain level, the larger molecular weight of PE caused the polymer chains to stack and fold, and due to the strong intermolecular forces, the coal tar became dispersed within the copolymer phase. Consequently, the impact of PE modification decreased [53]. At this point, polarized images indicated a decreasing trend in the content of the mesophase.

### 3.4. EVA-Modified Carbon Mesophase

#### 3.4.1. Modified with Different VA Content

**Figure 7:**
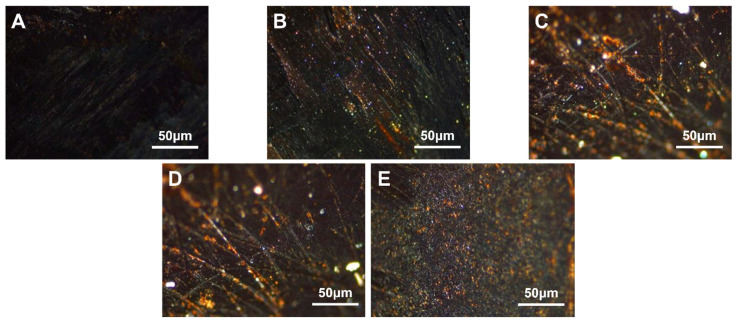
Polarization diagram of carbonaceous mesophase modified by EVA with five different amounts of VA (**A**)—MPE_1_, (**B**)—MPE_2_, (**C**)—MPE_3_, (**D**)—MPE_4_, (**E**)—MPE_5_).

Five types of EVA granules with varying VA content were selected. Coal tar, using heat, was mixed and stirred with 4.0 wt.% of EVA containing 12.0 wt.%, 18.0 wt.%, 25.0 wt.%, 32.0 wt.% and 40.0 wt.% VA, respectively. The resulting modified carbonaceous mesophase was designated as MPE_12_, MPE_18_, MPE_25_, MPE_32_ and MPE_40_. The polarized images magnified at a factor of 500× *g* are shown in Figure 7.

From Figure 7, it was observed that the asphalt prepared as the mesophase with EVA containing 40% VA as a copolymer exhibited a larger mesophase size. It could be inferred that with a higher VA content, the quantity of the resulting carbonaceous mesophase increased [54]. During the pyrolysis process, a substantial amount of ethylene vinyl acetate (EVA) hindered the coalescence of mesospheric microspheres, resulting in the significant growth of smaller spheres. Additionally, due to EVA’s higher melt index, its molecular mobility increased significantly upon surpassing the melting point temperature. This led to enhanced rheological properties and improved flexibility, ultimately promoting a more orderly flow in the mixed system and facilitating the orderly growth of the carbonaceous mesophase.

#### 3.4.2. Modified with Different EVA Additions

**Figure 8:**
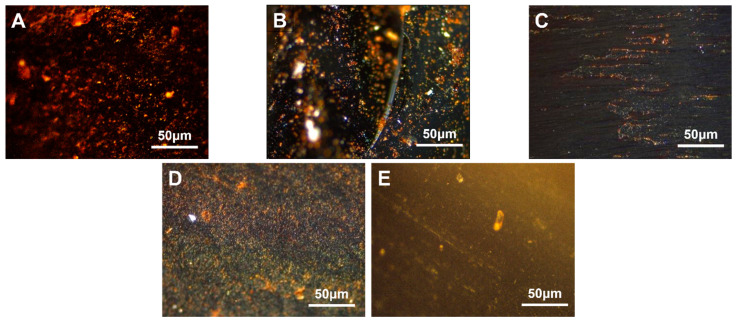
Polarization diagrams of mesophase pitch prepared with different amounts of VA40-EVA ((**A**)—MPE_40-2_, (**B**)—MPE_40-4_, (**C**)—MPE_40-6_, (**D**)—MPE_40-8_, (**E**)—MPE_40-10_).

High-density polyethylene was chosen as the modifier. Coal tar, using heat, was mixed and stirred with 2.0 wt.%, 4.0 wt.%, 6.0 wt.%, 8.0 wt.% and 10.0 wt.% of EVA containing 40.0 wt.% VA, respectively. The resulting modified carbonaceous mesophase was denoted as MPE_40-2_, MPE_40-4_, MPE_40-6_, MPE_40-8_ and MPE_40-10_. The polarized images, magnified 500× *g*, are shown in Figure 8.

From Figure 8, it was observed that when the VA content ranged from 2.0 wt.% to 6.0 wt.%, the carbonaceous mesophase exhibited better orderliness. At 8.0 wt.% VA content, a relatively higher content of the mesophase was prepared. However, at 4.0 wt.% VA content, the growth of the mesophase was more orderly. With an increase in the addition amount, the modified coal tar exhibited higher viscosity and a higher softening temperature. When the addition amount exceeded 6.0 wt.%, a phase transition in the EVA-modified coal tar dispersed system might have occurred. At lower addition amounts, EVA particles dispersed within the coal tar phase. As the addition amount increased to a certain level, the content of EVA increased in the system, gradually enhancing the inhibition of mesospheric microsphere coalescence. Consequently, the microspheres continued to grow in number. Therefore, the polarized images showed that as the EVA addition amount increased, the EVA modification effect strengthened continuously, resulting in an upward trend in the content of the mesophase.

### 3.5. Comparison of the Effect of Carbonaceous Mesophase Modified by Copolymer

**Figure 9:**
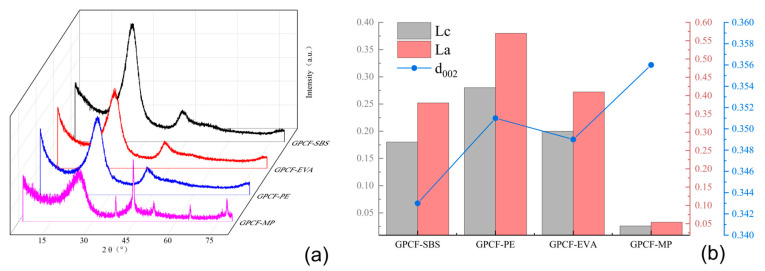
(**a**) XRD spectra of graphitized foam carbon prepared with different copolymer-modified carbonaceous mesophases; (**b**) preparation of graphitized foam carbon d_002,_ Lc and La from different copolymer-modified carbonaceous mesophases.

Using MPS_1301-10_, MPP_1-2_ and MPE_40-4_ as precursors, foam carbon materials were prepared and subsequently subjected to graphitization treatment with nickel metal as the catalyst. The graphitization treatment was employed as a catalyst. The resulting graphitized foam carbon samples were labeled as GPCF-SBS, GPCF-PE and GPCF-EVA, while graphitized foam carbon prepared from unmodified raw coal tar was designated as a blank control and labeled as GPCF-MP. Subsequently, the samples underwent XRD characterization to calculate d_002_, Lc and La values, with the results displayed in Figure 9.

From Figure 9a, it was observed that the graphitized foam carbons exhibited a distinct peak near 2θ = 26°. The peak for GPCF-MP was relatively broad with the lowest intensity, suggesting a lower degree of graphitization compared to the other three sample groups. GPCF-PE and GPCF-EVA also exhibited relatively broad peak profiles with lower diffraction intensities, indicating a lower degree of graphitization. In contrast, GPCF-SBS, prepared with 10% added SBS-1301 as the modifier, displayed a sharper peak profile with a higher diffraction intensity and relatively better symmetry, indicating a higher degree of graphitization.

By comparing the d_002_, Lc and La values of the four groups of graphitized foam carbons in Figure 9b, it was observed that three groups of foam carbons did not form a graphitic structure. In addition, the degree of proximity to a graphitic structure in the foam carbons could be analyzed based on the d_002_ value, which represents the (002) interlayer spacing. GPCF-MP had the most considerable d_002_ value at 0.356 nm, followed by GPCF-PE with a d_002_ value of 0.3515 nm. GPCF-EVA exhibited a d_002_ value of 0.3495 nm, making it the group of foam carbons closest to forming a graphitic structure among the three. GPCF-SBS had the smallest d_002_ value at 0.3430 nm among the three types of copolymer-based foam carbons, representing the only group that formed a graphitic structure. Its graphitization degree was calculated to be 10.5%, which was relatively low due to the lower temperature. The calculation of Lc and La values for the three types of graphitized foam carbons revealed that GPCF-SBS exhibited the largest Lc and La values, measuring 3.54 nm and 2.22 nm, respectively. These results aligned with the d_002_ analysis, indicating the most optimal formation of microcrystalline structures within the system. This verified the promoting effect of copolymers on the orderliness of the carbonaceous mesophase, and the promotion effect of three copolymer-modified mesophases on the graphitization degree of foam carbon decreased in the order of SBS-1301-10.0 wt.% > HDPE-2.0 wt.% > EVA-VA40-4.0 wt.%. This suggests that using 10.0 wt.% of SBS-1301 as a modifier to regulate the orderliness of the carbonaceous mesophase is the most suitable.

### 3.6. Analysis of the Modification Mechanism

#### 3.6.1. XRD Analysis

**Figure 10:**
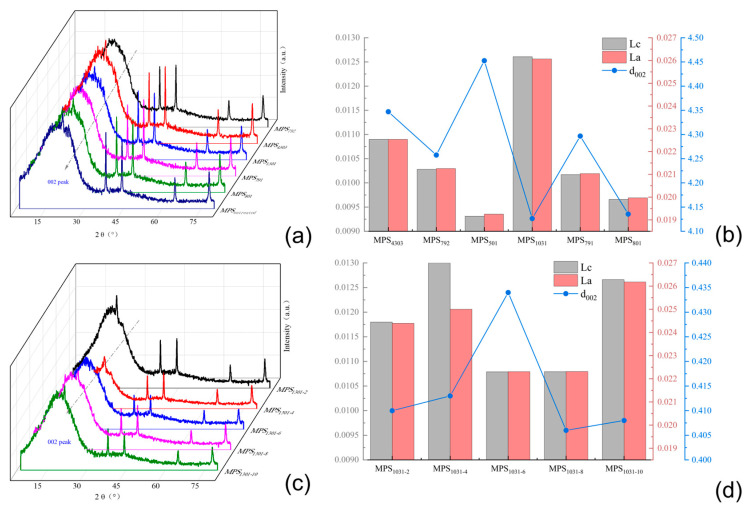
(**a**) XRD spectrogram analysis of carbonaceous mesophase with different SBS; (**b**) analysis of different SBS-modified carbonaceous mesophase d_002_, Lc and La; (**c**) XRD spectrogram analysis of modified coal-tar-based mesophase with different SBS-1301; (**d**) analysis of carbon mesophase d_002_, Lc and La modified by different SBS-1301 content.

For the five SBS-modified carbonaceous mesophases with a modifier addition of 4.0 wt.%, XRD analysis was conducted, and the calculated d_002_, Lc and La values are presented in Figure 10a and Figure 10b, respectively.

Figure 10a revealed the presence of both (002) crystal plane diffraction peaks and (100) crystal plane diffraction peaks in all six spectral lines near 2θ = 25° and 2θ = 43°. The sharpness and symmetry of these peak patterns served as measures of the degree of graphitization achieved in the sample. Nevertheless, since the carbonaceous mesophase served as an intermediate substance during the conversion of coal tar to carbon materials, it possessed a relatively disordered microcrystalline structure. Additionally, the solid carbonaceous mesophase contained a significant amount of impurities from heavy coal tar molecules. This resulted in less-than-optimal symmetry and relatively broad and flattened peak profiles observed in the (002) crystal plane diffraction peaks, as depicted in the figure. When comparing the XRD patterns of the mesophase obtained by direct thermal-polymerization of raw coal tar with that of the mesophase prepared after the addition of the copolymer SBS modifier, it was evident that the (002) crystal plane diffraction peak of the latter exhibited a sharper profile and better symmetry. MPS_1301_ and MPS_801_ were compared, and it was observed that MPS_1301_ exhibited a more pronounced impact on enhancing the carbonaceous mesophase. This was evident in the higher intensity, sharper profile and improved symmetry of the (002) crystal plane diffraction peak.

Figure 10b compares the values of d_002_, Lc and La for the five SBS-modified carbonaceous mesophases. Since the mesophase did not form a graphite-like structure, the system contained very few crystalline structures, resulting in a smaller microcrystalline stack thickness for Lc and a smaller microcrystal size for La. The comparison revealed that MPS_1301_ had the smallest d_002_ value among the five carbonaceous mesophases, indicating its closest proximity to a graphitic structure. Additionally, the Lc microcrystalline stack thickness and La microcrystal size were slightly higher in MPS_1301_ than in the other five carbonaceous mesophases. Hence, the SBS-1301-modified carbonaceous mesophase exhibited the highest degree of orderliness, confirming the superior promoting effect of SBS-1301 modification on the formation and ordered growth of the carbonaceous mesophase.

The modified carbonaceous mesophase with five different dosages of the SBS-1301 modifier was subjected to XRD analysis, and the calculated d_002_, Lc and La values are presented in Figure 10c,d.

In Figure 10c, five XRD patterns appear near 2θ = 25° and 2θ = 43°, exhibiting diffraction peaks corresponding to the (002) crystal plane and (100) crystal plane. In comparison, MPS_1301-10_ exhibited sharper peak profiles and better symmetry at the (002) crystal plane diffraction peak compared to MPS_1301-2_ and MPS_1301-4_. Additionally, it displayed a higher diffraction peak intensity at the (100) crystal plane. When comparing MPS_1301-4_, MPS_1301-6_, MPS_1301-8_ and MPS_1301-10_, it was observed that the intensity of the (002) crystal plane diffraction peak gradually decreased. Among them, MPS1301-10% exhibited the most substantial diffraction peak at the (100) crystal plane, indicating its highest aromatic condensation degree.

In Figure 10d, the d_002_ values, Lc and La of the five SBS-modified carbonaceous mesophase samples were compared. Among them, MPS_1301-6_ had the most considerable d_002_ value of 0.4336 nm, indicating its lowest degree of orderliness. The d_002_ value of MPS_1301-10_ was the smallest among the five different additive amounts, measuring 0.4084 nm, indicating its closest proximity to a graphitic structure. Meanwhile, the Lc and La values of MPS_1301-10_ were also slightly larger than those of the other four mesophases, and the trend was opposite to that of d_002_. Namely, the two values first decreased and then increased with the enhancement of the additive amount, which indicated that the degree of ordering of the carbonaceous mesophase did not increase with the increase in the additive amount of SBS-1301. Upon comparing the values, it was concluded that among the five additive amounts, the degree of orderliness in the modified carbonaceous mesophase was highest when the addition of SBS-1301 was 10%.

#### 3.6.2. FT-IR Analysis

**Figure 11:**
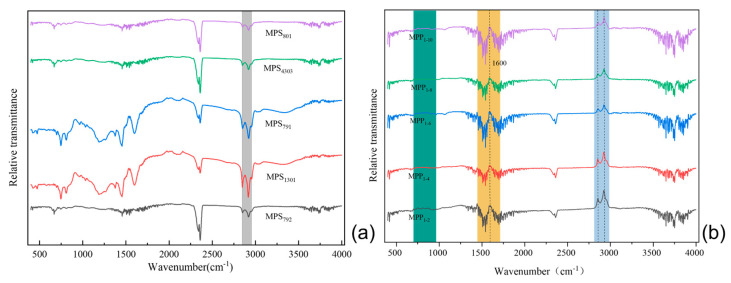
(**a**) FT−IR plot of the effect of different SBS modifications on the coal-tar-based mesophase; (**b**) FT−IR diagram of the impact of further SBS-1301 additions on the carbonaceous mesophase.

Figure 11a shows the infrared spectra of the mesophase prepared by the thermal polycondensation of other SBS-modified coal tars. The infrared spectra of the five SBS-modified carbonaceous mesophases exhibited two relatively intense aliphatic C–H stretching vibration peaks in the range of 2800 cm^−1^ to 3000 cm^−1^. When compared to the original tar infrared spectra, it was evident that the intensity of these stretching vibration peaks had significantly decreased, indicating the occurrence of aliphatic carbon chain structure fragmentation during the preparation of the carbonaceous mesophase [55]. At 1460 cm^−1^, a methylene absorption peak appeared, and in comparison, MP-1301 exhibited a nearly vanished absorption peak at this position. It was inferred that methyl and methylene free radicals acted as bridging connectors between aromatic rings during the generation process within the mesophase, facilitating the growth and orderly development of the mesophase. This indicated that SBS-1301 was more effective in promoting mesophase growth.

Figure 11b presents the FT−IR spectra, illustrating the influence of different SBS-1301 addition levels on the modified carbonaceous mesophase. All five groups of mesophases exhibited two relatively strong aliphatic C–H stretching vibration peaks in the range of 2800 cm^−1^ to 3000 cm^−1^. In contrast, the absorption peak for the 10.0 wt.% addition level was relatively weaker, suggesting that during the high-temperature reaction process, the thermal decomposition of aliphatic carbon chains led to chain scission. There were lower-intensity absorption peaks in the vicinity of 1600 cm^−1^, corresponding to the skeletal C=C stretching vibration of PAHs or condensed aromatic hydrocarbons. At 1380 cm^−1^, there was a low-intensity methyl C–H absorption vibration peak. At 1460 cm^−1^, there was a prominent methylene absorption peak. Interestingly, in the mesophase prepared with 10.0 wt.% SBS-1301 as the modifier, the intensity of this absorption peak was notably weaker. This observation suggests the formation of a methylene bridge structure between two aromatic rings during aromatization and thermal condensation processes. This phenomenon likely promoted the formation and orderly development of the carbonaceous mesophase, resulting in the generation of a greater quantity of larger-sized mesophase [56].

**Figure 12:**
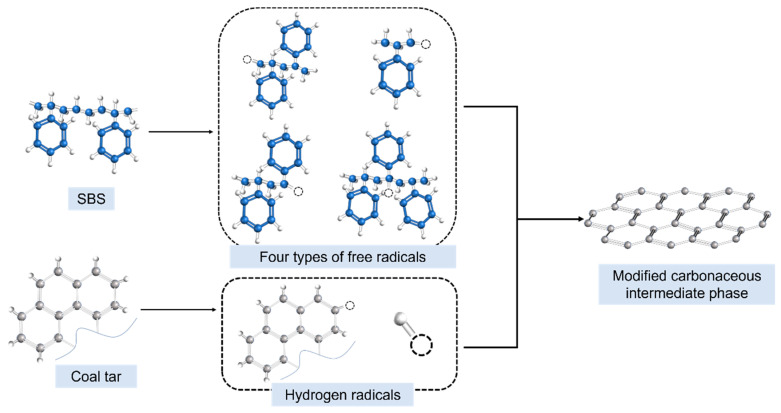
Mechanism diagram of SBS-modified preparation of carbonaceous mesophase.

As depicted in Figure 12, SBS dissolved in coal tar underwent chain scission and decomposition under high-temperature and high-pressure conditions, leading to the release of molecules such as styrene and butadiene. These styrene molecules could further generate methyl and methylene radicals, among others. The radicals produced from coal tar components interacted with those generated from styrene molecules, resulting in a significant population of methyl and methylene radicals in the reaction system. This reduced the affinity energy density of heavy coal tar molecules, enhancing the affinity of heavy tar. Consequently, it promoted the condensation polymerization and stacking of PAHs, significantly increasing the content of the soluble carbonaceous mesophase. Furthermore, the methyl and methylene radicals improved the fluidity of the system, facilitating collisions and coalescence among mesophase microspheres. This, in turn, led to the formation of larger-sized mesophases and enhanced the alignment of carbonaceous mesophases.

## 4. Conclusions

Compared to 2.0 wt.% HDPE and 4.0 wt.% EVA with a VA content of 40.0 wt.%, linear SBS with a styrene/butadiene ratio (S/B) of 30/70 at 10.0 wt.% is the most effective copolymer for the ordered modification of heavy coal tar. Furthermore, in comparison to HDPE-2.0 wt.% and EVA-VA40-4.0 wt.%, the foam carbon prepared with SBS-1301-10.0 wt.% as the polymer modifier is the only one among the three that forms a graphitic structure, with a d_002_ of 0.3430 nm and the maximum Lc and La values of 3.54 nm and 2.22 nm, respectively. This is attributed to the breakage of aliphatic carbon chain structures during the formation of the carbonaceous mesophase. Methyl and methylene radicals act as bridges between aromatic rings, facilitating collisions and fusion between the small mesophase spheres, thereby forming larger-sized mesophases. This promotes the growth and orderly development of the mesophase, resulting in a better-aligned orientation of the carbonaceous mesophase.

In summary, the copolymerization with 10.0 wt.% linear SBS with a styrene/butadiene ratio (S/B) of 30/70 is the most effective for the ordered modification of heavy coal tar to prepare mesophases with optimal orderliness. This contributes to the subsequent preparation of high-performance graphitic foam carbon.

## Figures and Tables

**Table 1:** Properties of coal tar.

HS (wt.%)	HI-TS (wt.%)	TI-QS (wt.%)	QI (wt.%)
92.28	6.88	0.84	-

Note: HS: n-hexane soluble fraction, HI-TS: n-hexane-insoluble–toluene-soluble fraction, TI-QS: toluene-insoluble but quinoline-soluble, QI: quinoline-insoluble.

**Table 2:** Heavy coal tar components.

Asphalt	Anthracene Oil	Wash Oil	Naphthalene Oil	Phenol Oil	Light Oil	Other
90.5%	3.4%	2.3%	1.8%	1.8%	0.1%	0.1%

**Table 3:** The grades and properties of SBS.

Brand	1301	4303	792	YH-801	791-H
S/B	30/70	30/70	40/60	30/70	30/70
Structure	Linear	Radial	Linear	Radial	Linear

**Table 4:** The specifications and properties of PE.

Category	High-Density Polyethylene	Linear Low-Density Polyethylene	Low-Density Polyethylene
Specification	HDPE	LLDPE	LDPE
Crystallinity (%)	85~97	50~55	55~65
Density (g/cm^3^)	0.941~0.960	0.918~0.935	0.91~0.93

**Table 5:** The grades and properties of EVA.

VA contents	12 wt.%	18 wt.%	25 wt.%	32 wt.%	40 wt.%
Melt index (g/10 min) (190 °C/2.16 kg)	8	8	19	43	52

## Data Availability

The data presented in this study are available on request from the corresponding author.
